# Impact of Obesity on the Association Between Salt Intake and Blood Pressure in Adult Men With Hypertension Who Participated in the Remote Lifestyle Intervention

**DOI:** 10.1155/jobe/1822308

**Published:** 2026-07-14

**Authors:** Takuji Adachi, Masashi Kanai, Takahiro Miki, Kotoe Shimizu, Michitaka Kato, Yuta Hagiwara

**Affiliations:** ^1^ Major in Rehabilitation Science, Department of Health Sciences, Nagoya City University Medical School, Nagoya, Aichi, Japan; ^2^ Institute of Transdisciplinary Sciences for Innovation, Kanazawa University, Kanazawa, Ishikawa, Japan, kanazawa-u.ac.jp; ^3^ Insight Lab, Prevent Inc., Nagoya, Aichi, Japan

**Keywords:** blood pressure, lifestyle modification, obesity, salt sensitivity

## Abstract

**Introduction:**

Experimental studies have demonstrated that obesity increases salt sensitivity, defined as the blood pressure (BP) response to salt intake; however, its relevance within real‐world lifestyle modification settings remains uncertain. This study aimed to investigate the cross‐sectional and longitudinal associations between daily salt intake and BP according to obesity status.

**Methods:**

This observational study analyzed data from adult men with hypertension who participated in a remote lifestyle modification program. Daily salt intake was estimated from urinary sodium excretion using a validated self‐monitoring device and expressed as equivalent sodium chloride (NaCl) (g/day), and home systolic blood pressure (SBP) was recorded. A cross‐sectional analysis compared SBP among the tertiles of salt intake at baseline. Longitudinal analysis was used to compare SBP reduction among the tertiles of salt intake reduction over 3 months. These analyses were adjusted for potential confounders and stratified by obesity, defined as body mass index ≥ 25 kg/m^2^.

**Results:**

A total of 1095 participants were included (median age, 57 years; obese, 67.2%). At baseline, higher daily salt intake was associated with higher SBP in the obesity group (lowest tertile, estimated mean; 130.7 mmHg [95% confidence interval 129.2 to 132.3]; middle tertile, 132.2 [130.7 to 133.8]; highest tertile, 133.2 [131.6 to 134.7]; *p* = 0.028), but not in the nonobese group (*p* = 0.174). After 3 months, a greater reduction in salt intake was associated with a greater SBP decrease in the obese group (lowest tertile, estimated mean −2.5 [−3.5 to −1.4] mmHg; middle tertile, −3.5 [−4.5 to −2.4] mmHg; highest tertile, −5.2 [−6.3 to −4.1] mmHg; *p* < 0.001) but not in the nonobese group (*p* = 0.335).

**Conclusion:**

These findings suggest that, in real‐world lifestyle modification settings, changes in salt intake are associated with changes in BP among adult men with obesity, highlighting the clinical relevance of salt sensitivity for tailored BP management.

## 1. Introduction

Obesity is a well‐established contributor to elevated blood pressure (BP), which is a leading cause of cardiovascular diseases. Obesity‐related hypertension has complex pathways involving increased circulating blood volume, kidney compression, obstructive sleep apnoea, and hormonal and metabolic changes caused by excess adipose tissue [1]. Among the mechanisms, hormonal and metabolic changes lead to increased renal sodium reabsorption and plasma volume along with increased vascular tone via elevated renin–angiotensin–aldosterone overactivity, sympathetic nervous system overactivity, insulin resistance, and vascular dysfunction [1]. This altered internal environment caused by obesity results in elevated salt sensitivity and BP response to changes in salt intake [2]. Therefore, salt intake reduction is acknowledged as a core component of lifestyle modification for BP control, particularly in individuals with obesity.

To date, the link between obesity and salt sensitivity has been investigated by examining the effects of weight loss interventions or comparing obese and healthy individuals. Prior intervention studies demonstrated that weight loss decreases salt sensitivity, and a BP lowering effect of weight loss is prominent in salt‐sensitive individuals [3–5]. Another study reported that metabolic syndrome, defined as a combination of abdominal obesity, elevated BP, impaired glucose metabolism, and dyslipidemia, is associated with a high probability of salt sensitivity [6]. This relationship is supported by a recent observational study showing that a greater number of metabolic risk factors were associated with higher renin–angiotensin–aldosterone activity and salt sensitivity in obese individuals [7]. These results suggest that weight loss attenuates the increase in BP induced by salt intake, whereas salt intake reduction likely effectively decreases BP in obese individuals.

However, previous findings cannot be directly applied to lifestyle modification programs because of the experimental protocols for assessing salt sensitivity. In the conventional method, the difference in BP during one week on a high‐salt diet (e.g. 12–15 g/day) and another week on a low‐salt diet (e.g. 1–3 g/day) was calculated as an index of salt sensitivity [2, 8]. The low‐salt intake controlled by these experimental protocols is not realistically achievable outside of the research setting, and such a rapid change in salt intake cannot occur in real‐world lifestyle modifications. Such an extreme reduction in salt intake has not been achieved, even in previous interventional studies of reducing salt intake [9, 10]. Therefore, it remains uncertain whether the effect of changes in daily salt intake on home BP varies depending on the presence of obesity.

Therefore, this study aimed to examine the association between salt intake and BP based on the presence of obesity using cross‐sectional and longitudinal analyses of hypertensive adult men who participated in a remote lifestyle modification program.

## 2. Methods

### 2.1. Study Design and Participants

This retrospective observational study used data from Prevent Inc. (Nagoya, Japan), a company that provides medical data analysis and mobile app‐based lifestyle modification support programs for patients with atherosclerotic risk factors. Commissioned by the Health Insurance Association, Prevent Inc., provides a mobile app–based lifestyle modification support program called MyStar to individuals at high risk of cerebrovascular and cardiovascular diseases. Prevent Inc. predicted the risk of cardiovascular and cerebrovascular disease onset using health insurance claims and health checkup data to select a high‐risk population expected to reduce disease risk through lifestyle modifications. Employees or their dependents who are at high risk of onset can be provided with an individualized support program using Mystar. Japanese law requires business operators to conduct annual health checkups on their employees. Hence, the study participants had standard insurance coverage and did not undergo additional health checkups.

Mystar targets individuals with hypertension, diabetes, and dyslipidemia, who are taking medications or have a history of coronary artery disease or stroke. A list of diagnoses (including arrhythmia, cardiomyopathy, end‐stage kidney disease, and mental disorders) and medications (including cardiotonic, immunosuppressive, and antipsychotic agents) prohibiting participation in the Mystar program is available at GitHub (https://github.com/PREVENT-Inc/MyscopeMasterList).

The inclusion criteria for the present analysis were adult men with hypertension who participated in a mobile app–based lifestyle modification program. The present analysis excluded women because of the potential sex‐specific differences in salt sensitivity [11] and the limited sample size of women participants in our database (approximately 15%). Other exclusion criteria were participants who dropped out of the program and those with missing data. This study analyzed data from individuals who participated in the program between December 2018 and November 2023.

This study was performed following the principles of the Declaration of Helsinki and approved by the Research Ethics Committee of Nagoya University (approval no. 2022‐0453) and the Research Ethics Committee of Nagoya City University Graduate School of Nursing (approval no. 25023). The participants agreed to a privacy policy at the start of the program, stating that the data gathered in the app may be used for future research. Participants were given the right to refuse to participate at any time (opt‐out option). Information regarding this study, including the inclusion criteria and the opt‐out option, was available on the website of Prevent Inc. The study protocol was approved by our ethics committee. None of the participants had opted out of the study at the time of analysis.

### 2.2. Mobile App–Based Lifestyle Modification Program

The lifestyle modification support program was implemented with the approval of attending physicians. The objective of the program was to control risk factors including hypertension, diabetes, and dyslipidemia through lifestyle improvements to prevent the onset of atherosclerotic diseases such as stroke and myocardial infarction. Participants were provided with a mobile app account to participate in the Mystar program, which allowed them to record their lifestyle habits and exchange chat messages with their assigned healthcare professionals on their smartphones. The participants were also provided with a self‐monitoring device to measure salt intake and a wrist‐worn activity tracker (Fitbit Inspire, Fitbit Inc.) to measure step count.

The first 2 weeks of the intervention focused on understanding the importance of lifestyle self‐monitoring and familiarity with the Fitbit and monitoring device for salt intake and self‐recording of lifestyle on the Mystar app. The program lasted 6 months, during which all participants had 12 telephone consultations with healthcare professionals and biweekly feedback via chat messages. Nurses, registered dietitians, physical therapists, and other healthcare professionals provided individual advice based on participants’ lifestyles and disease management conditions via phone calls and chat messages.

The participants set behavioral goals during telephone consultations for the next 2 weeks and worked to improve lifestyle habits, such as physical activity and diet, by recording them using a mobile app. Lifestyle information recorded on the application was stored as lifelogs that participants and healthcare professionals could review at any time. Participants were able to ask healthcare professionals questions using a mobile app, and healthcare professionals provided individual advice on behavioral changes. Further details of the program are provided below [12, 13].

### 2.3. Daily Salt Intake Measurement

Daily salt intake was estimated using a self‐monitoring device (GENEN Monitor, Kono ME Institute, Kawasaki, Japan). This device estimates salt intake from urinary sodium excretion and expresses the results as equivalent sodium chloride (NaCl) intake (g/day). The measurement is based on urine conductivity, which is used to estimate NaCl concentration, and a validated formula is applied to estimate 24‐h salt excretion from overnight urine samples. The reliability and validity of this device have been previously reported [14].

Urine collection over a 24‐h period is considered the most reliable method for evaluating salt intake [15]. However, because of the difficulty in collecting complete and accurate 24‐h urine samples from employees, this method is not feasible for self‐monitoring of daily salt intake. In such cases, alternative methods are commonly used in clinical practice. The Japanese Society of Hypertension recommends estimating daily salt intake using nighttime urine, the second voided urine after waking (which can be used with the Kawasaki formula [16]), or spot urine samples (commonly used with the Tanaka formula [17]) as practical substitutes for 24‐h urine collection [18].

The device used in this study estimates 24‐h salt excretion based on overnight urine samples, and the correlation between measured 24‐h urinary salt excretion and the estimated value has been reported to be 0.72 [14]. Participants were instructed to void completely and discard any urine obtained before going to bed. They were then required to collect all urine produced during the night and the first voided urine after waking, corresponding to approximately 8 h of sleep. A 1‐L cup was provided for this purpose, and the measurement device was placed into the collected urine sample to assess conductivity and urine volume. Participants manually entered the estimated salt intake (g/day) value displayed on the device into the mobile application. In this study, the mean values of daily salt intake for consecutive 7 days at baseline and at 3 months of the program were calculated for the analysis. Weekly mean values were calculated using ≥ 3 days of measurements, which have been shown to provide reasonable estimates compared with 24‐h urine collection methods [14]. The mean daily salt intakes calculated by ≤ 2 days were considered missing data, and the participants were excluded from the analysis.

### 2.4. Home BP Measurement

Participants were instructed to measure their home BP according to the current guidelines [19]. In Japan, almost all individuals with hypertension have their own standard home BP monitoring devices [20]. In this study, the participants used their own devices based on the cuff‐oscillometric method using an upper arm cuff that followed domestic standards and obtained medical device approval from the Ministry of Health, Labour, and Welfare of Japan.

In this study, morning BP was measured to minimize the effects of antihypertensive medications, physical activity during the day, and diet. Morning BP was measured within 1 h after waking up, after urination, before dosing in the morning, before breakfast, and after 1‐2 min resting in a sitting position [19]. The participants measured their BP twice, and the mean value of the two measurements was recorded on the app. In this study, the mean values of morning systolic BP for consecutive 7 days at baseline and at 3 months of the program were calculated for the analysis. The mean BP for each week was calculated using ≥ 5 days according to the current guidelines [19]. BP measurements ≤ 4 days were considered missing data, and the participants were excluded from the analysis.

### 2.5. Participant Characteristics

Demographic information, including age, sex, height, and weight at baseline, was obtained directly from the participants at the start of the program. Body mass index (BMI) was calculated by dividing the weight (kg) by height squared (m^2^). To assess physical activity, all participants were provided with a wrist‐worn activity tracker (Fitbit Inspire, Fitbit Inc.) and instructed to self‐monitor their activity. The mean daily step count over one week at baseline was used in the analysis. The validity of Fitbit‐derived step counts has been demonstrated in previous studies comparing them with established accelerometers [21].

Medical history information for each participant was collected from the claims data provided by the insurers before the start of the program. In Japan, medical receipts describing the medical treatments administered to patients and the corresponding fees are collected monthly by health insurance unions. Therefore, this study included participants from the general health insurance system in Japan and did not target populations enrolled in special or nonstandard insurance programs.

### 2.6. Statistical Analysis

The Shapiro–Wilk test was used to assess the normal distribution of the data. Continuous variables were presented as mean (standard deviation [SD]) or median (interquartile range [IQR]) in cases of non‐normal distribution. Categorical variables are presented as percentages to describe participant characteristics. Baseline characteristics were compared between participants with and without obesity using the Mann–Whitney *U* test for continuous variables and the chi‐square test for categorical variables. In addition, changes in BP and salt intake over the 3‐month intervention period were compared across BMI categories (< 25.0, 25.0–34.9, and ≥ 35.0 kg/m^2^) using the Kruskal–Wallis test.

In the present study, we conducted (1) a cross‐sectional analysis to examine the association between salt intake and BP, and (2) a longitudinal analysis to examine the relationship between the change in salt intake and the reduction in BP for 3 months. The longitudinal analysis was conducted over a 3‐month period because behavioral changes were expected to occur within the first two to three months of the program [22]. Additionally, a longer follow‐up period could generate various confounding factors, including medication changes and significant weight loss in those with obesity at baseline during the program, and relapse in lifestyle behavior in the second half of the program. To this end, we aimed to avoid the influence of these factors by analyzing the 3‐month period but not the whole 6‐month intervention period.

In the cross‐sectional analysis at baseline, we first evaluated the association between baseline morning systolic BP and tertiles of salt intake in the overall population using linear regression analysis. The model was adjusted for potential confounders, including age, obesity status (BMI ≥ 25 kg/m^2^), daily step count, use of antihypertensive agents, diabetes mellitus, and dyslipidemia. In addition, we tested the interaction between salt intake and obesity status. Given the limited sample size, a two‐sided *p* value < 0.10 was considered to indicate a potential interaction. Subsequently, we performed stratified analyses according to obesity status. Baseline morning systolic BP across the tertiles of salt intake in those with and without obesity was compared using analysis of variance (ANOVA), followed by analysis of covariance (ANCOVA) adjusted for the same covariates except obesity status.

In the longitudinal analysis from baseline to 3 months, we first examined the association between changes in salt intake (tertiles) and changes in BP in the overall population using linear regression analysis. The model was adjusted for age, obesity status, daily step counts, use of antihypertensive agents, diabetes mellitus, and dyslipidemia. We also tested the interaction between changes in salt intake and obesity status by including an interaction term in the model. Subsequently, we performed stratified analyses according to obesity status. Changes in BP across tertiles of salt intake reduction were compared using ANOVA, followed by ANCOVA.

Except for interaction tests, statistical significance was set at *p* < 0.05. All analyses were performed using the Stata software (Version 18.0; StataCorp LP, College Station, TX, USA).

## 3. Results

**FIGURE 1:**
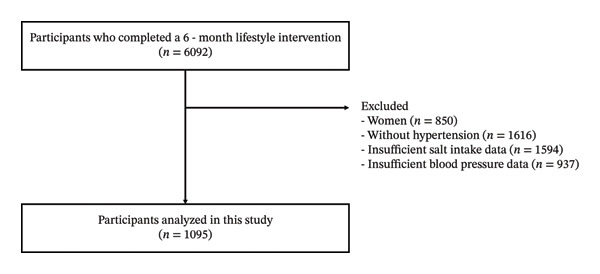
Participant selection flowchart.

This study included 1095 participants of the remote intervention (Figure 1). The participants’ characteristics are presented in Table 1. The median age was 57 (IQR 53–61) years, and the median BMI was 26.4 (IQR 24.2–29.3) kg/m^2^ (prevalence of obesity: 67.2%). The median morning systolic and diastolic BPs at baseline were 131 (IQR 124–138) mmHg (mean 131.0; SD 11.5) and 85 (IQR 79–90) mmHg (mean 84.9; SD 8.7), respectively. The obesity group was younger and had a higher BMI, higher prevalence of diabetes mellitus, lower prevalence of a history of stroke, higher BP, greater salt intake, lower daily step counts, greater use of antihypertensive medications, and higher levels of HbA1c and triglycerides compared with the nonobesity group. Changes in BP and daily salt (NaCl‐equivalent) intake over the 3‐month intervention period across BMI categories were also summarized in Supporting Table 1.

**TABLE 1:** Characteristics of study participants.

	Overall (*n* = 1095)	Nonobesity (*n* = 359)	Obesity (*n* = 736)	*p*
Age, years	57 (53–61)	59 (54–63)	57 (52–61)	< 0.001
Body mass index, kg/m^2^	26.4 (24.2–29.3)	23.3 (22.2–24.2)	28.3 (26.4–30.8)	< 0.001
Diabetes mellitus, %	38.2%	30.4%	42.0%	< 0.001
Dyslipidemia, %	57.6%	54.3%	59.2%	0.122
History of stroke, %	5.2%	7.5%	4.1%	0.016
History of ischemic heart disease, %	8.3%	9.7%	7.6%	0.228
Systolic blood pressure, mmHg	131 (124–138)	129 (122–136)	131 (124–139)	< 0.001
Diastolic blood pressure, mmHg	85 (79–90)	83 (79–88)	85 (80–91)	< 0.001
Daily salt intake, g/day	10.3 (8.6–12.2)	9.7 (8.1–11.6)	10.5 (8.9–12.4)	< 0.001
Step count, steps/day	9583 (7089–12085)	9981 (7408–17473)	9400 (8983–11801)	0.001
ACEi/ARB, %	53.7%	46.8%	57.1%	0.001
Calcium channel blocker, %	54.6%	49.6%	57.1%	0.001
Beta blocker, %	6.4%	5.3%	6.9%	0.299
Diuretic, %	6.3%	4.7%	7.1%	0.136
HbA1c, %	6.0 (5.5–6.7)	5.8 (5.4–6.4)	6.1 (5.6–6.8)	< 0.001
LDL‐C, mg/dL	120 (99–140)	116 (97–139)	121 (101–142)	0.070
TG, mg/dL	131 (93–190)	109 (79–156)	140 (102–206)	< 0.001
eGFR, mL/min/1.73m^2^	69.7 (60.9–78.8)	69.3 (60.9–77.9)	69.9 (60.8–79.0)	0.519

*Note:* Continuous variables are presented as median (interquartile range).

Abbreviations: ACEi, angiotensin‐converting enzyme inhibitor; ARB, angiotensin II receptor blocker; eGFR, estimated glomerular filtration rate; LDL‐C, low‐density lipoprotein cholesterol; TG, triglyceride.

In overall participants, morning systolic BP at baseline was significantly different across the tertiles of salt intake (lowest, mean 129.5 (SD 11.6) mmHg; middle, 130.6 (11.3); highest, 132.8 (11.4); *p* for trend < 0.001). In the linear regression analysis, higher salt intake was independently associated with higher baseline systolic BP, and obesity was also significantly associated with higher BP, whereas no significant interaction between salt intake and obesity was observed (Table 2).

**TABLE 2:** Results of linear regression analysis for blood pressure at baseline.

	Model 1			Model 2			Model 3		
	Regression coefficient	95% CI	*p*	Regression coefficient	95% CI	*p*	Regression coefficient	95% CI	*p*
Salt intake, lowest tertile	(Reference)			(Reference)			(Reference)		
Intermediate	1.01	−0.64–2.68	0.230	0.79	−0.87–2.46	0.350	0.78	−1.16–2.74	0.430
Highest tertile	3.32	1.66–4.98	< 0.001	2.69	1.00–4.38	0.002	2.60	0.01–5.25	0.048
Age, per 1 year	0.08	−0.22–0.19	0.120	0.11	0.01–0.22	0.032	0.13	0.01–0.24	0.027
Obesity, yes				2.93	1.49–4.38	< 0.001	3.02	−0.43–6.47	0.086
Step count, per 1000 steps/day				−0.06	−0.22–0.11	0.509	−0.01	−0.18–0.16	0.868
Antihypertensive agents, yes				−0.75	−2.28–0.79	0.341	−0.80	−2.37–0.78	0.321
Diabetes mellitus, yes				−0.39	−1.80–1.02	0.584	−0.20	−1.66–1.23	0.786
Dyslipidemia, yes				−1.51	−2.89–−0.13	0.032	−1.73	−3.18–−0.30	0.018
Salt intake ∗ obesity (interaction)							0.35	−1.48–2.18	0.707

*Note:* Dependent variable, morning systolic blood pressure at baseline.

Abbreviation: CI, confidence interval.

**FIGURE 2:**
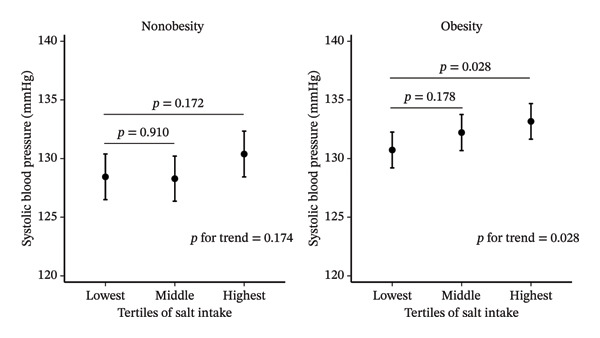
Comparisons of baseline morning systolic blood pressure among the tertiles of daily salt intake.

In the obesity group, greater salt intake was associated with higher BP (lowest, mean 130.4 (SD 12.5) mmHg; middle, 132.0 (11.1); highest, 133.5 (11.7); *p* for trend = 0.015); this relationship was not observed in the nonobese group (lowest, mean 128.5 (SD 9.5) mmHg; middle, 128.2 (11.4); highest, 130.3 (10.6); *p* for trend = 0.227). In the multivariate analysis, this trend remained after adjusting for potential confounders in the obesity group (lowest tertile, estimated mean; 130.7 mmHg [95% confidence interval 129.2 to 132.3]; middle tertile, 132.2 [130.7 to 133.8]; highest tertile, 133.2 [131.6 to 134.7]; *p* for trend = 0.028, whereas this was not observed in the nonobese group (*p* = 0.174) (Figure 2).

After 3 months, the participants’ morning systolic BP decreased by a mean of 3.3 ± 8.3 mmHg. In overall participants, BP reduction was significantly different across the tertiles of salt intake reduction (lowest, mean −2.6 (SD 8.4) mmHg; middle, −2.8 (7.8); highest, −4.4 (8.8); *p* for trend = 0.004). In the linear regression analysis, greater salt reduction was independently associated with a larger decrease in systolic BP. A significant interaction between salt reduction and obesity status was observed. In models without the interaction term, obesity at baseline was also associated with greater BP reduction (Table 3).

**TABLE 3:** Results of linear regression analysis for blood pressure reduction for 3 months.

	Model 1			Model 2			Model 3		
	Regression coefficient	95% CI	*p*	Regression coefficient	95% CI	*p*	Regression coefficient	95% CI	*p*
Salt reduction, lowest tertile	(Reference)			(Reference)			(Reference)		
Intermediate	−0.28	−1.49–0.93	0.649	−0.27	−1.48–0.94	0.664	0.21	−1.34–1.77	0.789
Highest tertile	−1.97	−3.16–−0.74	0.002	−1.97	−3.19–−0.76	0.002	−1.46	−2.91–−0.01	0.049
Age, per 1 year	−0.01	−0.08–0.08	0.986	−0.01	−0.08–0.07	0.947	0.01	−0.08–0.09	0.911
Obesity, yes				−1.30	−2.35–−0.25	0.015	0.44	−2.48–3.36	0.768
Step count, per 1000 steps/day				−0.04	−0.15–0.09	0.583	−0.02	−0.14–0.11	0.763
Antihypertensive agents, yes				−1.19	−2.30–−0.07	0.036	−1.37	−2.51–−0.24	0.018
Diabetes mellitus, yes				−0.87	−1.91–0.16	0.098	−1.06	−2.14–0.01	0.052
Dyslipidemia, yes				0.74	−0.27–1.75	0.153	0.88	−0.18–1.93	0.104
Salt reduction ∗ obesity (interaction)							−1.05	−2.28–0.18	0.094

*Note:* Dependent variable, reduction in morning systolic blood pressure for 3 months.

Abbreviation: CI, confidence interval.

**FIGURE 3:**
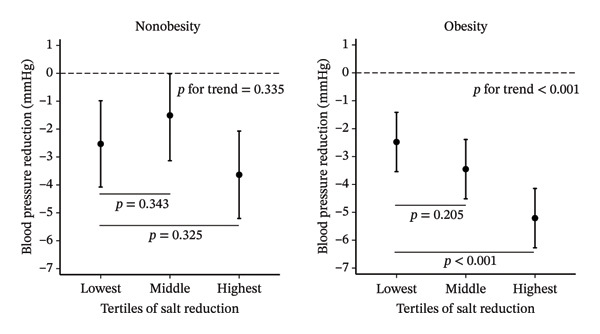
Comparisons of morning systolic blood pressure reduction for 3 months among the tertiles of daily salt intake reduction.

In the obesity group, greater salt reduction was associated with decrease in BP (lowest, mean −2.9 (SD 8.0) mmHg; middle, −3.7 (8.5); highest, −4.4 (8.3); *p* for trend = 0.047); this relationship was not observed in the nonobese group (lowest, mean −3.3 (SD 8.4) mmHg; middle, −1.7 (8.2); highest, −2.6 (8.8); *p* for trend = 0.332). In multivariate analysis, this trend remained after adjusting for potential confounders in the obesity group (lowest tertile, estimated mean −2.5 mmHg [95% confidence interval −3.5 to −1.4]; middle tertile, −3.5 [−4.5 to −2.4]; highest tertile, −5.2 [−6.3 to −4.1]; *p* for trend < 0.001), but this was not observed in the nonobese group (*p* = 0.335) (Figure 3).

## 4. Discussion

In this study, we examined the association between salt intake and BP in daily living among the participants of a remote lifestyle modification program. Salt intake was associated with BP, and this relationship was greater in obese individuals. These findings from the real‐world data analysis are aligned with previous experimental studies, which indicate that obesity leads to elevated salt sensitivity. This suggests that weight loss, combined with salt reduction, may contribute to BP control in obese individuals with hypertension, along with other lifestyle factors.

Salt sensitivity, defined as the degree of BP response to changes in salt intake, varies considerably among individuals [23, 24]. Identifying individuals with high salt sensitivity helps tailor lifestyle interventions to improve BP control. Increased salt sensitivity is linked to poor hypertension management and a higher risk of cardiovascular disease [25, 26]. It affects approximately 25% of the general population and up to 50% of patients with essential hypertension. Unlike animal models, the human BP response to salt intake is highly variable and is influenced by genetic and environmental factors [27]. Many genetic variants related to renal sodium handling and hormonal regulation have been associated with salt sensitivity [28–31], and heritability has been estimated to be approximately 50% in previous familial studies [32, 33]. Genetic factors strongly influence salt sensitivity; however, they alone do not fully explain this condition. Previous studies have shown that age, sex, birth weight, and body weight are associated with salt sensitivity [27] and that obesity and metabolic syndrome are key modifiable factors that may increase it [2]. Identifying such modifiable contributors could help plan personalized strategies for BP control.

The findings of this study extend previous experimental evidence suggesting enhanced salt sensitivity in individuals with obesity [3–7] and demonstrate its potential applicability in real‐world lifestyle modification programs. We investigated whether baseline salt intake and its reduction over a three‐month period were associated with BP among men participating in a mobile app–based remote lifestyle intervention. Importantly, the longitudinal association was primarily observed among obese participants. Although previous studies assessed salt sensitivity using highly controlled protocols involving extreme modifications in dietary salt intake, which are difficult to replicate in routine care, our study evaluated gradual and naturally occurring changes in salt intake resulting from lifestyle improvements. This approach better reflects real‐world practices. Given that obesity is associated with neurohormonal and metabolic disturbances that promote sodium retention and vascular dysfunction, our results are biologically plausible and underscore the clinical relevance of obesity‐related salt sensitivity. However, changes in salt intake may not solely reflect intentional salt reduction but could also represent broader modifications in overall dietary patterns. Therefore, the observed associations between salt intake and BP may partly capture the combined effects of multiple dietary factors. To isolate the independent effect of salt intake, further analyses incorporating detailed dietary assessments, including total energy intake, would be necessary.

To further clarify the role of obesity in our findings, we additionally examined its independent association with BP outcomes. In the present study, obesity was associated with higher baseline systolic BP, consistent with the well‐established role of excess body weight as a major determinant of hypertension [1]. Interestingly, obesity at baseline was also associated with a greater reduction in BP over the 3‐month intervention period. This finding may reflect a greater potential for improvement through lifestyle modification among individuals with obesity, who often present with multiple modifiable risk factors. Although no significant interaction between salt intake and obesity was observed in the cross‐sectional analysis at baseline, the longitudinal analysis suggested that greater salt reduction was associated with a larger decrease in BP among individuals with obesity. These findings indicate that, while obesity itself is an important determinant of BP, it may also modify the extent to which changes in salt intake translate into BP reduction over time.

Incorporating salt sensitivity into lifestyle interventions may also facilitate behavioral changes in individuals with obesity. Elevated BP is caused by multiple modifiable factors including excess salt intake, physical inactivity, alcohol consumption, and obesity. The Japan Atherosclerosis Society Guidelines for Cardiovascular Prevention recommend weight loss in obese individuals [34]. In general, a weight loss of approximately 3% over 3–6 months is recommended to avoid excess short‐term weight reduction [34]. Therefore, the physiological benefits of weight reduction on cardiovascular risk factors tend to gradually appear. As self‐efficacy and expectations are important factors in the promotion of behavioral changes, early experiences of BP reduction through salt reduction may enhance motivation and adherence to long‐term lifestyle changes. Individuals receiving treatment for cardiovascular risk factors often show a high level of interest in the mechanisms through which medications and lifestyle modifications prevent or improve disease regardless of their health literacy [35]. Therefore, communication tailored to health status and lifestyle is a key component of effective interventions.

This study had some limitations. First, the study population consisted only of men, which may limit the generalizability of the findings. In addition, sex differences in salt sensitivity could not be evaluated, warranting further studies including women [11]. Second, although the home BP monitors used by the participants met domestic regulatory standards, the models were not standardized among the participants. Although standardizing devices for all participants is impractical in routine lifestyle interventions, this heterogeneity may have affected the precision of BP estimation. Third, detailed dietary data, including total energy intake, were not available. Therefore, the independent effect of salt intake could not be fully distinguished from other dietary factors. Fourth, changes in BP medications during follow‐up could not be considered because of lack of data. Finally, owing to the observational nature of this study, causal relationships between salt intake, weight loss, and BP changes could not be established.

## 5. Conclusions

The association between salt intake and BP was more evident in the obese group, cross‐sectionally and longitudinally in real‐world lifestyle modification settings. These findings highlight the potential clinical relevance of salt sensitivity and support the importance of obesity‐specific, tailored strategies for BP management.

## Author Contributions

Takuji Adachi contributed to the conception, study design, analysis, and interpretation of data and drafted the manuscript for this work. Kotoe Shimizu and Takahiro Miki contributed to the data acquisition and interpretation. Masashi Kanai contributed to the interpretation of the data. Yuta Hagiwara coordinated and supervised the project, and interpreted the data.

## Funding

This work was supported by JSPS KAKENHI (grant number 23K16769) and the Taiju Life Social Welfare Foundation.

## Disclosure

All the authors critically revised the manuscript, approved the final version, and agreed to be accountable for all aspects of the study, thereby ensuring its integrity and accuracy.

## Conflicts of Interest

Prevent, Inc. provides an mHealth‐based disease management program. Takahiro Miki and Kotoe Shimizu are employees of Prevent, Inc. Masashi Kanai has received consulting fees from Prevent Inc. and is a nonregular staff member. Yuta Hagiwara is the founder and Chief Executive Officer of Prevent Inc. Takuji Adachi and Michitaka Kato declare no conflicts of interest.

## Supporting Information

Additional supporting information can be found online in the Supporting Information section.

## Supporting information


**Supporting Information** Supporting Table 1. Systolic blood pressure and salt intake according to obesity status.

## Data Availability

The data underlying this article cannot be shared publicly due to the privacy of individuals that participated in the study. The data will be shared on reasonable request to the corresponding author.
