# Supplementary material for: Impact of Obesity on the Association Between Salt Intake and Blood Pressure in Adult Men With Hypertension Who Participated in the Remote Lifestyle Intervention
Source: J Obes. 2026 Jul 14;2026:1822308. doi: 10.1155/jobe/1822308 (PMC13366498; doi:10.1155/jobe/1822308)
Supplement: Supplementary file 1 — Supporting Information Supporting Table 1. Systolic blood pressure and salt intake according to obesity status. [file JOBE-2026-1822308-s001.docx]

**Supplementary materials**

**Supplementary Table 1.** Systolic blood pressure and salt intake according to obesity status

|  | BMI <25.0 kg/m^2^  (n=359) | BMI 25.0-34.9 kg/m^2^  (n=686) | BMI ≥35.0 kg/m^2^  (n=50) | p |
| --- | --- | --- | --- | --- |
| Systolic blood pressure at baseline, mmHg | 129 (122–136) | 131 (124–139) | 135 (127–143) | <0.001 |
| Systolic blood pressure at 3-month, mmHg | 126 (119–134) | 128 (120–136) | 130 (126–139) | 0.011 |
| Blood pressure reduction for 3 months, mmHg | -2.5 (-7.3–2.4) | -3.0 (-8.4–1.8) | -2.9 (-8.3–0.1) | 0.217 |
| Salt intake at baseline, g/day | 9.7 (8.1–11.6) | 10.4 (8.9–12.2) | 12.8 (10.5–15.2) | <0.001 |
| Salt intake at 3-month, g/day | 9.2 (7.9–10.8) | 9.8 (8.2–11.5) | 11.3 (9.6–12.8) | <0.001 |
| Salt reduction for 3 months, g/day | -0.6 (-2.1–0.7) | -0.5 (-2.0–1.0) | -1.0 (-2.6–0.1) | 0.025 |

BMI, body mass index

Data are presented as median (interquartile range).
